# Exploring the Bidirectional Causal Pathways Between Smoking Behaviors and Headache: A Mendelian Randomization Study

**DOI:** 10.1093/ntr/ntad173

**Published:** 2023-09-13

**Authors:** Katherine Lloyd, Sean Harrison, Hannah M Sallis, George Davey Smith, Marcus R Munafò, Robyn E Wootton

**Affiliations:** Department of Population Health Sciences, Bristol Medical School, University of Bristol, Bristol, UK; Department of Population Health Sciences, Bristol Medical School, University of Bristol, Bristol, UK; MRC Integrative Epidemiology Unit, Bristol, UK; Department of Population Health Sciences, Bristol Medical School, University of Bristol, Bristol, UK; MRC Integrative Epidemiology Unit, Bristol, UK; School of Psychological Science, University of Bristol, Bristol, UK; Department of Population Health Sciences, Bristol Medical School, University of Bristol, Bristol, UK; MRC Integrative Epidemiology Unit, Bristol, UK; School of Psychological Science, University of Bristol, Bristol, UK; NIHR Bristol Biomedical Research Centre, University Hospitals Bristol NHS Foundation Trust and University of Bristol, Bristol, UK; MRC Integrative Epidemiology Unit, Bristol, UK; School of Psychological Science, University of Bristol, Bristol, UK; NIHR Bristol Biomedical Research Centre, University Hospitals Bristol NHS Foundation Trust and University of Bristol, Bristol, UK; MRC Integrative Epidemiology Unit, Bristol, UK; School of Psychological Science, University of Bristol, Bristol, UK; Nic Waals Institute, Lovisenberg Diaconal Hospital, Oslo, Norway

## Abstract

**Introduction:**

Although observational data suggest a relationship between headache and smoking, there remain questions about causality. Smoking may increase headache risk, individuals may smoke to alleviate headaches, or smoking and headache may share common risk factors. Mendelian randomization (MR) is a method that uses genetic variants as instruments for making causal inferences about an exposure and an outcome.

**Aims and Methods:**

First, we conducted logistic regression of observational data in UK Biobank assessing the association between smoking behaviors (smoking status, cigarettes per day amongst daily smokers, and lifetime smoking score) on the risk of self-reported headache (in the last month and for more than 3 months). Second, we used genetic instruments for smoking behaviors and headache (identified in independent genome-wide association studies [GWAS]) to perform bidirectional MR analysis.

**Results:**

Observationally, there is a weak association between smoking behavior and experiencing headache, with increased cigarettes per day associated with increased headache risk. In the MR analysis, genetic liability to smoking initiation and lifetime smoking increased odds of headache in the last month but not odds of headaches lasting more than 3 months. In the opposite direction, there was weak evidence for higher genetic liability to headaches decreasing the chance of quitting.

**Conclusions:**

There was weak evidence for a partially bidirectional causal relationship between smoking behaviors and headache in the last month. Given this relationship is distinct from smoking heaviness, it suggests headache and smoking may share common risk factors such as personality traits.

**Implications:**

Using MR, this study addresses the uncertainty regarding the observed relationship between headache and smoking. There was evidence for weak causal effects of smoking initiation and lifetime smoking (but not smoking heaviness) on likelihood of experiencing headache in the last month, but not over a prolonged period of more than 3 months. Those with higher genetic liability to headaches were also less likely to successfully stop smoking. This partially bidirectional causal relationship distinct from smoking heaviness suggests that observed associations are unlikely due to biological effects of tobacco smoke exposure and may be explained by shared personality traits.

## Introduction

The worldwide prevalence of headache disorder is 47%,^1^ and it is rated the third highest cause of disability worldwide.^2^ Headache-related consultations account for 44 per 1000 patients in primary care, costing £956 million per year in the United Kingdom.^3,4^ The huge personal and societal costs have encouraged researchers and clinicians to explore possible modifiable risk factors. Numerous observational studies in small populations suggest an association between headache and smoking,^5,6^ more specifically, migraine^7^ and cluster headache.^8–10^ This has been replicated in large population-level data.^11–14^ Notably in the HeadHUNT study, there was a linear trend of increasing likelihood of headache with increasing cigarettes per day in >40 000 adults.^11^

Hypothesized mechanisms involve the effect of cigarette smoke on the central nervous system, and theories differ for each primary headache disorder. For cluster headache, cadmium-induced neurotoxicity, trigeminal autonomic reflex activation, and nicotine-mediated hormone modulation have all been postulated as mechanisms.^15^ As migraine patients have a greater risk of early-onset stroke and a less favorable cardiovascular risk profile, it is hypothesized that smoking increases the risk of migraine via its vascular consequences.^16^ Smoking is associated with multiple chronic pain conditions, and an altered pain phenotype, through mechanisms such as receptor sensitization from chronic nicotine exposure, could also be implicated in headache.^17–19^

**Figure 1.**
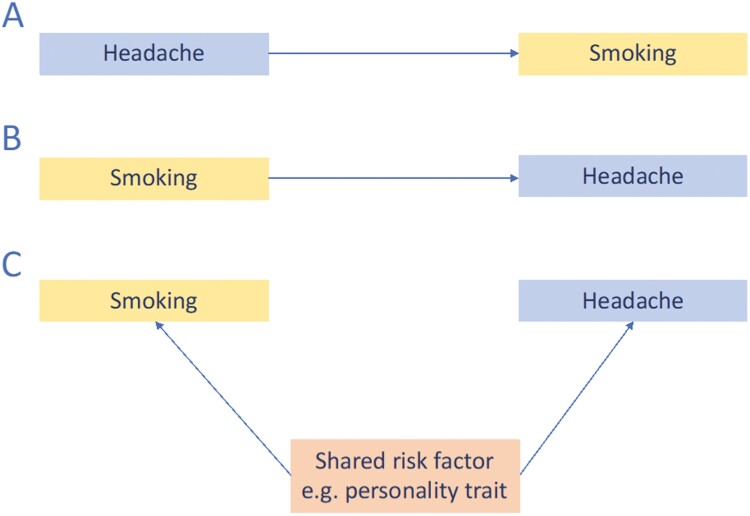
Directed acyclic graphs of possible nature of the observed relationship between headache and smoking. (A) Headache leads to initiation of smoking, for example, as a coping mechanism. (B) Smoking causes headache, for example, via neurotoxicity or pain modulation. (C) A common shared risk factor (eg, a personality trait that predisposes to both increased risk of headache and of altered behaviors such as initiating smoking).

Given the observational nature of data on smoking and headache, there remain questions about causality. It can be hypothesized that smoking causes the increased risk of headache, or that individuals with frequent headaches smoke in order to alleviate pain by way of a coping mechanism or as self-medication.^19^ Alternatively, there might be no causal effect, but headache and smoking might share common risk factors (Figure 1). One approach to strengthening causal inference is through Mendelian randomization (MR),^20^ which can be implemented through instrumental variable (IV) analysis, with genetic variants as the IVs for potentially modifiable exposures.^21^ MR makes use of naturally occurring quasi-randomization of genetic variants in the population, which typically reduces bias from confounding factors. Associations with genotype are extremely unlikely to be affected by reverse causation as genotype is stable across the lifetime. MR studies can be conducted in large population-based samples, increasing the power and generalizability of results. Through the use of genetic instruments of liability to exposures, an MR study can also overcome the ethical barriers to implementing harmful randomized interventions such as smoking.

Two previous one-sample MR studies did not find robust evidence for a causal effect of smoking intensity on headache.^22,23^ However, we now have new genetic instruments that provide greater power and include smoking initiation^24^ as well as lifetime smoking exposure (combining smoking duration, heaviness, and cessation).^25^ These instruments also allow for two-sample MR, which has greater power and can better explore potential bias from directional horizontal pleiotropy via a range of sensitivity analyses. We have conducted a two-sample MR analysis using GWAS summary data, with the aim to explore whether the associations between smoking and headache are causal.

## Methods

### Observational Analysis

#### Sample

We used data from the UK Biobank, a national health resource comprising individuals aged 40–69 years who were registered with the National Health Service and living up to about 25 miles from a study assessment center, and were invited to participate. Between 2006 and 2010, 503 325 individuals were recruited and provided various measures including physical and mental health.^26^ Genotyping procedure and quality control are described elsewhere.^27^

#### Measures of Smoking Behavior

At recruitment, participants self-reported measures of smoking status (current, former, never) and smoking heaviness (number of cigarettes per day among daily smokers). UK Biobank excluded responses fewer than 1 cigarette per day or more than 150 cigarettes per day. Loose tobacco smokers were told that “One ounce of tobacco makes about 30 cigarettes and one gram of tobacco makes about 1 cigarette.” All responses of “prefer not to say” or “don’t know” were coded as missing. Our analysis of smoking initiation compared ever smokers (current + former smokers) with never smokers. Our analysis of smoking cessation compared current smokers with former smokers. Lifetime smoking is a continuous composite measure of smoking initiation, heaviness, duration, and cessation, described in detail elsewhere.^25^

#### Measures of Headache

We derived two measures of headache. The first captures any experience of headache in the past month. Participants were asked about headaches as part of a question on pain (data field 6159). They were asked “In the last month have you experienced any of the following that interfered with your usual activities?” and one of the options was headaches along with pain in other specified body areas. Participants were able to select multiple options. Individuals responding positively to headache were coded as 1 and participants responding “none of the above” or any other pain excluding headache were coded as 0. Participants responding “Prefer not to answer” were coded as missing. If individuals selected the “headache” response, they were asked a follow-up question, “Have you had headaches for more than 3 months?” (data field 3799). We used this to derive a second measure of headaches lasting longer than 3 months, where participants responding “yes” were coded as 1, and anyone responding “no” or being a 0 for headaches in the last month were both coded as 0.

#### Statistical Analysis

After restricting individuals of European ancestry with genetic data and headache measures available (to make this analysis comparable with subsequent analyses), 336 441 participants remained. We looked at the effect of four smoking behaviors: smoking status (ever vs. never), smoking status (current vs. former within ever smokers), cigarettes per day (within ever daily smokers), and lifetime smoking score. The latter is a combination of smoking duration, smoking cessation, and smoking heaviness described in detail elsewhere.^25^ The association between each of these smoking behaviors on risk of being troubled by headaches was estimated using logistic regression, controlling for birth year, sex, socioeconomic position (SEP), alcohol consumption, sleep duration, body mass index (BMI), allergic conditions, and health care seeking for mental distress. Each of these covariate measures is described in detail in Supplementary Note. All analyses were conducted using R^28^ and statistical codes are available from the authors upon reasonable request.

### Mendelian Randomization Analysis Using Summary-Level Data

MR utilizes genetic variants as instrumental variables to estimate the causal effect of an exposure (eg, smoking) on an outcome (eg, headache). The method of (MR makes three core assumptions: (1) the relevance assumption—the genetic instrument must be robustly associated with the exposure; (2) the independence assumption—the genetic instrument must not be associated with confounders of the exposure–outcome relationship; and (3) the exclusion–restriction assumption—the genetic instrument must only be associated with the outcome via the exposure.^20^ The latter two assumptions can be violated by horizontal pleiotropy, which occurs when the genetic instruments for the exposure are also associated with confounders, or the outcome directly. We use a range of sensitivity analyses (outlined below) to explore the likelihood of assumption violations.

We looked at the effect of two measures of smoking behavior (smoking initiation and lifetime smoking) on headaches because these measures do not require stratification into smokers and nonsmokers (which is not possible using summary-level data). All GWAS were of European Ancestry and conducted on adults to ensure similarity between the exposure and outcome populations. We also explored the effect of headache liability on smoking behaviors (initiation, lifetime smoking, heaviness, and cessation) to assess possible reverse causation.

### GWAS Summary Statistics

#### Smoking Initiation

The smoking initiation (ever vs. never) instrument was taken from the latest GWAS conducted by the GSCAN consortium.^29^ Smoking initiation was defined as having smoked more than 100 cigarettes or ever having smoked regularly, and the GWAS controlled for age, age squared, sex, and genetic principal components.^29^ After being restricted to the European ancestry-only subsample, and performing clumping at 1000 kb and *r*^2^ < 0.001, there were 248 independent genome-wide significant single nucleotide polymorphisms (SNPs) available (see Supplementary Data Sheet). Due to sharing agreements, our summary statistics do not include the 23andMe sample, leaving a sample size of 805 431 individuals.

#### Lifetime Smoking on Headache

The GWAS of lifetime smoking was conducted in 462 690 individuals of European ancestry from the UK Biobank.^25^ The instrument identified was 126 genome-wide significant SNPs, clumped to ensure independence at 1000 kb and *r*^2^ < 0.001 (see Supplementary Data Sheet).

#### Headache at 1 Month

Meng et al.^30^ conducted a GWAS in 223 773 individuals (74 461 cases and 149 312 controls) from the UK Biobank, using the same question as detailed above “In the last month have you experienced any of the following that interfered with your usual activities?,” where those who selected “headache” were defined as cases and those who had selected “none of the above” or any other pain response were the controls. The GWAS controlled for age, sex, BMI, nine population principal components, genotyping arrays, and assessment centers as covariates.^30^ They identified 28 loci associated with genome-wide significance, of which 14 had previously been associated with migraine.^30,24^

#### Headache at 3 Months

Using the measure of headaches experienced for more than 3 months (detailed above), we conducted our own GWAS using the MRC Integrative Epidemiology Unit GWAS pipeline.^31^ We used BOLT LMM (which accounts for population stratification using linear mixed modeling) controlling for sex and genotyping chip. We restricted to SNPs with MAF < 0.01 and INFO > 0.8. The final sample size was 335 059, of which there were 30 017 cases and 305 042 controls. We identified 17 genome-wide significant SNPs, clumped to ensure independence at 1000 kb and *r*^2^ < 0.001 (see Supplementary Data Sheet).

#### Smoking Heaviness

We used the most recent GWAS of smoking heaviness conducted by the GSCAN consortium.^29^ Due to sharing agreements, our summary statistics do not include the 23andMe sample, leaving a sample size of 326 497 individuals after restricting to those of European ancestry. Smoking heaviness was defined as number of cigarettes smoked per day among both current and former smokers collapsed into binned categories: 1–5, 6–15, 16–25, 26–35, and ≥36 cigarettes per day.

#### Smoking Cessation

We used the most recent GWAS of smoking cessation conducted by the GSCAN consortium,^29^ which compared current to former smokers. Due to sharing agreements, our summary statistics do not include the 23andMe sample, leaving a sample size of 388 313 individuals after restricting to those of European ancestry.

### Statistical Analysis

All analyses were conducted using the TwoSampleMR package, version v0.4.17^32^ for R.^28^ We used five different MR methods: inverse-variance weighted, MR Egger,^33^ weighted median,^34^ weighted mode,^35^ and MR RAPS.^36^ Each method makes different assumptions about the presence of pleiotropy and therefore a consistent effect across multiple methods strengthens causal evidence. If an SNP was unavailable in the outcome GWAS summary statistics, then proxy SNPs were searched for with a minimum linkage disequilibrium *r*^2^ = 0.8, and palindromic SNPs were aligned if minor allele frequency (MAF) < 0.3. Where exposures were binary, effect estimates were multiplied by 0.693 so that units can be interpreted as a change per doubling in the odds of the exposure.^37^ We estimated instrument strength by calculating the mean *F*-statistic across all SNPs, where an *F*-statistic < 10 is considered weak.^38^ We calculated the regression dilution *I*^2^_GX_ as an indicator of the suitability of the instrument for MR Egger. When *I*^2^_GX_ was between 0.6 and 0.9, a SIMEX correction was performed.^38^ We performed Rucker’s *Q* test of heterogeneity and the MR Egger intercept test to estimate potential directional horizontal pleiotropy.^33^ We performed Steiger filtering to check all genetic variants explained more variance in the exposure than the outcome. If this is not the case, it could suggest potential reverse causation.^39^ Scatter plots and leave-one-out SNP plots were visually inspected to check for possible outliers.

There was an overlap between our exposure and outcome samples with UK Biobank contributing to both. Although recent evidence suggests that bias from sample overlap is minimal when sample sizes are large,^40^ we conducted a sensitivity analysis using MRlap to assess whether there was substantial bias from sample overlap between our exposure and outcome samples.^41^ Finally, we replicated our analysis using a FinnGen GWAS of “other headache syndromes,” which excludes migraines but includes other headache subtypes including cluster headaches.^42^ See Supplementary Note for details.

### Single SNP Analysis of rs1051730

#### Statistical Analysis

Best-guess genotypes at the SNP rs1051730 were extracted using Plink^43^ in the UK Biobank full sample described above. rs1051730 is in the gene cluster *CHRNA5-A3-B4* and is known to be strongly associated with heaviness of smoking.^44^ We tested using logistic regression whether the number of effect alleles (A) of this SNP was associated with the risk of being troubled by headaches using the same measure as described above. We controlled for age, sex, and 10 principal components of population structure in all analyses. The logistic regressions were run in each category of smoking status separately (ever, current, former, and never smokers). A causal effect of smoking on the risk of headaches would be characterized by an effect of rs1051730 in all categories of smoking status apart from never smoking which provides the negative control.

## Results

### Observational Analysis

After restricting to individuals of European ancestry who passed genetic exclusions^27^ and had completed the measure of pain in the last month, 336 441 individuals remained. The mean age was 56.9 years (SD = 8.00) and 54% were female; 45% had ever smoked, 10% were current smokers, and 35% were former smokers. Logistic regressions of self-reported smoking behavior on experiencing headaches in the last month generally showed very weak evidence of an association with small effect sizes (Table 1). Smoking more cigarettes per day was associated with an increased risk of headaches in the last month and an increased risk of headaches for more than 3 months. Conversely, a higher lifetime smoking score was associated with a decreased risk of headaches for more than 3 months. Finally, current smoking (vs. quitting) was associated with a decreased risk of experiencing headaches for more than 3 months.

**Table 1.** Observational associations between measured smoking behaviors and experience of headaches

	*N*	OR (95% CI)	*p*
Headache in the last month			
Smoking Initiation (ever vs. never)	329 229	0.99 (0.98, 1.01)	.55
Smoking cessation (current vs. former)	148 632	1.01 (0.98, 1.04)	.60
Cigarettes per day (per cigarette)	97 969	1.003 (1.00, 1.004)	.003
Lifetime smoking (per 1 point increase on lifetime smoking index^28^)	329 244	0.99 (0.98, 1.01)	.37
Headache for more than 3 months			
Smoking initiation (ever vs. never)	327 954	0.99 (0.97, 1.02)	.54
Smoking cessation (current vs. former)	148 046	0.94 (0.90, 0.99)	.01
Cigarettes per day (per cigarette)	97 562	1.003 (1.00, 1.005)	.02
Lifetime smoking (per 1 point increase on lifetime smoking index^28^)	327 969	0.98 (0.96, 0.99)	.008

Adjusted for sex, birth year, socioeconomic position, alcohol consumption, sleep duration, body mass index, allergies, and mental distress (see Supplementary Note for details).

### Mendelian Randomization Analysis Using Summary Data

Harmonized SNP-exposure and SNP-outcome effects are presented in Supplementary Data Sheet. We found evidence to support an effect of genetic liability to smoking initiation and lifetime smoking on increased odds of headache in the last month with small effect sizes (Table 2). The direction of effect was consistent across all methods, which each make different assumptions about pleiotropy. There was weaker evidence from the weighted mode method, but this method has the lowest power. However, there was no evidence to suggest that genetic liability to smoking initiation and lifetime smoking increased odds of experiencing headaches for more than 3 months (Table 2). The measure of regression dilution *I*^2^_GX_ was below 0.6 for smoking initiation (see Supplementary Table 2); therefore, MR Egger analysis was not conducted.^33^ The regression dilution *I*^2^_GX_ was below 0.9 for lifetime smoking (Supplementary Table 2); therefore, an unweighted MR Egger SIMEX correction was applied (Table 2).^38^ The Rucker’s *Q*-statistic suggested evidence of heterogeneity (Supplementary Table 3) but the MR Egger intercept showed no evidence for directional pleiotropy (Supplementary Table 4). We conducted Steiger filtering to check the direction of effect, and 100% of the SNPs explained more variance in the assumed exposure than the assumed outcome, suggesting bias from reverse causation is unlikely^39^ (Supplementary Table 5). MRlap suggested that estimates were minimally biased by sample overlap (Supplementary Table 7). Results were relatively consistent when replicating using the FinnGen cohort (see Supplementary Table 1 and Supplementary Note).

**Table 2.** Two-sample Mendelian randomization analyses of the effect of smoking initiation on risk of headaches

Exposure	Outcome	Method	*N* SNPs	OR/Beta (95% CI)	*p*
Smoking initiation	Headache in the last month	Inverse-variance weighted	217	1.032 (1.012, 1.051)	.001
		MR Egger	-	-	-
		Weighted median	217	1.021 (1.001, 1.042)	.04
		Weighted mode	217	1.029 (0.98, 1.08)	.25
		MR RAPS	217	1.032 (1.012, 1.052)	.001
Lifetime smoking	Headache in the last month	Inverse-variance weighted	120	1.08 (1.04, 1.12)	<.001
		MR Egger (SIMEX)	120	1.12 (1.03, 1.23)	.01
		Weighted median	120	1.07 (1.03, 1.10)	<.001
		Weighted mode	120	1.08 (0.98, 1.18)	0.13
		MR RAPS	120	1.07 (1.04, 1.11)	<.001
Smoking initiation	Headache >3 months	Inverse-variance weighted	236	1.001 (0.992, 1.009)	.88
		MR Egger	-	-	-
		Weighted median	236	0.998 (0.988, 1.008)	.68
		Weighted mode	236	0.997 (0.97, 1.026)	.86
		MR RAPS	236	1.001 (0.992, 1.009)	.85
Lifetime smoking	Headache >3 months	Inverse-variance weighted	120	1.02 (1.00, 1.03)	.05
		MR Egger (SIMEX)	120	1.05 (1.01, 1.09)	.008
		Weighted median	120	1.01 (1.00, 1.03)	.09
		Weighted mode	120	1.02 (0.97, 1.06)	.46
		MR RAPS	120	1.02 (1.00, 1.03)	.06

All effect estimates with binary exposures (smoking initiation) have been multiplied by 0.693, so that they can be interpreted as change in the outcome per doubling in the odds of the exposure. Lifetime smoking = a continuous composite measure of smoking initiation, heaviness, duration, and cessation, standardized. One-SD increase in lifetime smoking score is, for example, equivalent to being a current smoker who has smoked five cigarettes per day for 12 years, or a former smoker who smoked five cigarettes per day for 21 years but stopped smoking 10 years ago, rather than a never smoker. All outcomes are binary and units are odds ratios.

In the opposite direction, we did not find evidence to suggest that a genetic liability to increased risk of headache was associated with initiating smoking, nor smoking heaviness, with inconsistent directions of effect (Table 3). There was weak evidence for genetic liability to both headache measures decreasing the chance of quitting with consistent direction of effect and consistency in effect estimates across all sensitivity methods (with the exception of MR SIMEX) but estimates were imprecise (Table 3). For each analysis with headache as the exposure, regression dilution *I*^2^_GX_ was below 0.9 (Supplementary Table 2); therefore, an unweighted MR Egger SIMEX correction was applied (Table 3).^38^

**Table 3.** Two-sample Mendelian randomization analyses of the effect of headache risk on smoking behaviors

Exposure	Outcome	Method	*N* SNPs	OR/Beta (95% CI)	*p*
Headache last month	Smoking initiation	Inverse-variance weighted	27	1.04 (0.92, 1.17)	.55
		MR Egger (SIMEX)	27	0.99 (0.79, 1.25)	.98
		Weighted median	27	1.01 (0.93, 1.09)	.84
		Weighted mode	27	1.00 (0.92, 1.08)	.99
		MR RAPS	27	1.03 (0.92, 1.15)	.63
Headache >3 months	Smoking Initiation	Inverse-variance weighted	17	0.90 (0.74, 1.10)	.29
		MR Egger (SIMEX)	17	1.29 (0.74, 2.23)	.39
		Weighted median	17	0.95 (0.82, 1.10)	.47
		Weighted mode	17	0.97 (0.81, 1.17)	.75
		MR RAPS	17	0.94 (0.79, 1.13)	.53
Headache last month	Smoking heaviness	Inverse-variance weighted	27	0.093 (−0.145, 0.331)	.45
		MR Egger (SIMEX)	27	−0.005 (−0.330, 0.319)	.97
		Weighted median	27	−0.001 (−0.166, 0.164)	.99
		Weighted mode	27	−0.024 (−0.243, 0.195)	.83
		MR RAPS	27	0.018 (−0.186, 0.222)	.86
Headache >3 months	Smoking heaviness	Inverse-variance weighted	17	−0.034 (−0.256, 0.188)	.77
		MR Egger (SIMEX)	17	0.476 (0.130, 0.822)	.02
		Weighted median	17	0.048 (−0.273, 0.368)	.77
		Weighted mode	17	0.132 (−0.443, 0.706)	.66
		MR RAPS	17	−0.004 (−0.235, 0.228)	.98
Headache last month	Smoking cessation	Inverse-variance weighted	27	0.93 (0.86, 1.01)	.09
		MR Egger (SIMEX)	27	0.81 (0.63, 1.04)	.25
		Weighted median	27	0.95 (0.88, 1.04)	.26
		Weighted mode	27	0.94 (0.85, 1.04)	.24
		MR RAPS	27	0.94 (0.86, 1.02)	.11
Headache >3 months	Smoking cessation	Inverse-variance weighted	17	0.91 (0.80, 1.02)	.11
		MR Egger (SIMEX)	17	0.97 (0.65, 1.47)	.12
		Weighted median	17	0.93 (0.79, 1.11)	.43
		Weighted mode	17	0.93 (0.76, 1.13)	46
		MR RAPS	17	0.91 (0.80, 1.03)	0.14

All effect estimates have binary exposures (headache in the last month, headache for more than 3 months) so have been multiplied by 0.693, so that they can be interpreted as change in the outcome per doubling in the odds of the exposure. Lifetime smoking = a continuous composite measure of smoking initiation, heaviness, duration, and cessation, standardized. One-SD increase in lifetime smoking score is, for example, equivalent to being a current smoker who has smoked five cigarettes per day for 12 years, or a former smoker who smoked five cigarettes per day for 21 years but stopped smoking 10 years ago, rather than a never smoker. When outcomes are binary, units are odds ratios. When smoking heaviness is the outcome, units are betas (units of smoking heaviness are standardized bins of cigarettes per day, see Methods for further details).

### Single SNP Analysis on Odds of Headache

**Figure 2.**
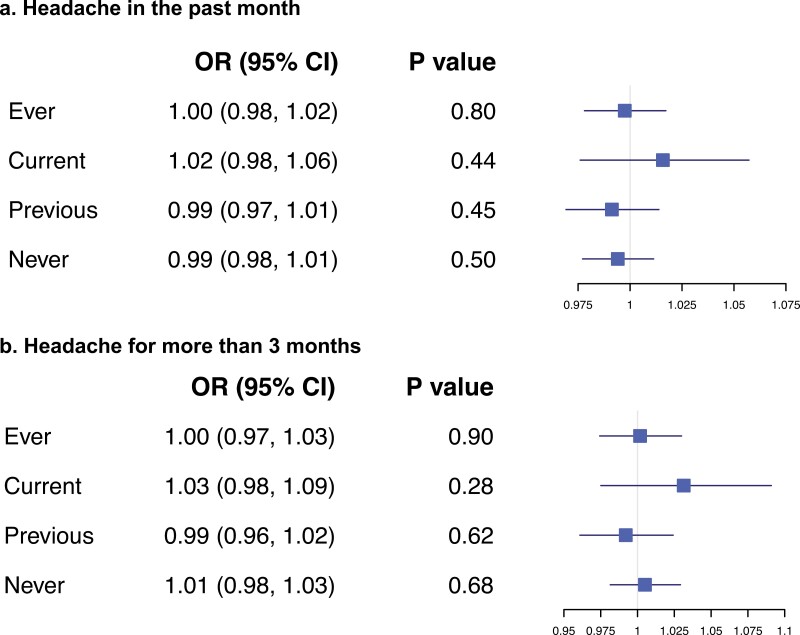
Single SNP Logistic regression results of genotype at rs1051730 on odds of experiencing headache split by smoking status. Units are per rs1051730 allele increase.

We looked at genotype at the SNP rs1051730 known to be associated with increased heaviness of smoking stratified by smoking status and controlling for age and sex. If there is an effect of heaviness of smoking on odds of experiencing headaches, we would expect to see an effect in the smokers but not in the nonsmokers who serve as a negative control. We did not find evidence of an effect of rs1051730 in any of the smoking groups (Figure 2). We demonstrated the expected effect of rs1051730 on cigarettes per day with ~1 cigarette per day increase per allele increase (Supplementary Table 6) and tested for association with known confounders of smoking. There was some evidence to suggest that genotype was associated with education status and age in the ever smokers (Supplementary Table 6).

## Discussion

The aim of this study was to understand if the association between smoking and headache is causal or due to residual confounding. Using MR, we found weak evidence for a small causal effect of genetic liability to smoking initiation and lifetime smoking on risk of headache in the last month but little evidence for a dose–response relationship using genetic liability to increased smoking heaviness. The effect of smoking initiation and lifetime smoking was not seen when looking at headache persisting for more than 3 months. This contradicts our observational results, which show an association between current smoking, lifetime smoking, and cigarettes per day on headache risk, but no association with smoking initiation. In the reverse direction, we found no clear evidence of an effect of genetic liability to headache on increasing risk of smoking initiation or lifetime smoking. However, in those who have smoked, there was weak evidence that genetic liability to headaches decreased the odds of quitting.

We provide evidence for a causal relationship between a genetic liability to smoking initiation and lifetime smoking with experiencing headaches, distinct from smoking heaviness. Our results, therefore, do not support previous large observational studies concluding that heaviness of smoking increases the prevalence of headache in a dose-dependent manner.^11,45^ Instead, our results are consistent with a previous MR study of smoking heaviness, which found no evidence of a causal association with headache.^22^ This could suggest that previous observational studies have been biased by residual confounding. We also show consistency with a previous longitudinal observational study which found no temporal relationship between adolescent smoking and adulthood headaches in 980 subjects.^46^ This demonstrated causal relationship, distinct from smoking heaviness, suggests that the increased risk of headache is not related to the inhalation of smoke or cigarette contents but rather to behaviors related to smoking. One explanation for this is that the genetic instrument for smoking initiation and lifetime smoking confers certain personality traits that underpin both smoking-related behaviors and experiencing headaches. This is further supported by the partly bidirectional nature of the association where, in the other direction, a genetic liability to headaches confers a low likelihood of cessation, another smoking behavior. Certain personality traits have been well described in smoking and drug initiation including impulsivity^47^ and risk-taking.^48^ There are also reported associations between personality and migraine,^49^ cluster headache,^50^ and tension headache,^51^ including neuroticism, eccentric, obsessive–compulsive, and anxious behaviors.^52^ Although the role of psychosocial factors has been proposed in the complex relationship between smoking and pain,^19^ there are, to our knowledge, no studies exploring the common personality or behavior traits of headache and smoking. Such traits could include poor coping mechanisms of which headache and smoking initiation are both consequences, or personality types predisposing to headache and altering subsequent health-seeking behaviors including smoking cessation.

One explanation for the observational relationship seen between smoking and headache is that people who experience headaches may smoke by way of a coping mechanism.^19^ Our findings refute this hypothesis given we show no clear evidence of increased smoking in individuals with a genetic liability to headaches. An important finding to highlight is that we did not find an effect of genetic liability to smoking initiation/lifetime smoking on headache persisting for more than 3 months. This somewhat contradicts our other findings and could be explained in several ways. First, we do not know how valid a question this is; it may have been interpreted as a continuous headache for 3 months, meaning the episodic nature of all primary headache disorders was not validly captured. On the contrary, it could suggest that more chronic headache conditions such as migraine, etc., which you would expect to persist in an episodic way for more than 3 months, are not linked to the smoking behaviors and instead have a distinct pathophysiology. Finally, it could be the case that we were underpowered to detect the effects of this outcome, given that persistence for more than 3 months was substantially rarer than experiencing any headache in the past month.

This is one of few studies using MR that has explored whether there is a causal relationship between smoking behavior and headache. We used the most recent instruments for smoking initiation and lifetime smoking allowing us to conduct two-sample MR and therefore maximize sample size and power. These instruments along with the SNP rs1051730 allowed us to examine multiple facets of smoking behavior, and triangulate results across a number of research methods. We conducted multiple sensitivity analyses to test for bias from directional pleiotropy and there was no evidence of bias.

Given the prevalence, symptomology, management, and proposed pathophysiology of different headache disorders are distinct,^53^ a major limitation of large observational data is that headache subtype is often undifferentiated. Furthermore, the headache question referring to the experience of headache in the last month may have positively identified people who experienced a personally rare event of a headache and missed individuals with chronic headache who may have had a month without headache or been successfully controlled with treatment. We hope that our second headache measure, asking whether individuals have experienced headaches for 3 months or more, will better capture more chronic experiences of headaches. However, individuals were only asked to respond to this item if they had experienced headache in the last month, again potentially missing individuals who had been unusually headache free. Furthermore, rarer primary headache disorders, such as cluster headache which have been consistently linked to smoking,^15^ are likely to be poorly represented, even in the FinnGen replication sample, where all headache diagnoses were included. The small effect size seen could be explained by this heterogeneity of headache included, masking a larger effect of a specific headache subtype. When considering specific headache disorders our results should be interpreted with caution. Additionally, both UK Biobank headache measures were retrospective self-reports which could bias results, although estimates were relatively consistent when replicating in the FinnGen cohort which used ICD-10 registry codes.

UK Biobank data are further limited by selection bias, impacting the generalizability of the results. Participants are aged 39–70 years, are of European ancestry, are less likely to smoke, more likely to be highly educated and are overall healthier than the general population.^54^ There is observational evidence that the most significant increase in the prevalence of headache among smokers is in people under 40 years of age.^11,45^

Finally, our analysis of smoking heaviness stratified by smoking status could be impacted by collider bias. This is because there is evidence for an association between the genetic variant and smoking status in the UK Biobank.^23^ Consequently, any confounding variables that are associated with both smoking status and headache liability will open up an alternate path between the SNP and headache when we condition on smoking status. Collider bias could make it difficult to estimate the true association between the SNP and headache risk. However, previous simulations of this collider bias in the UK Biobank suggested that the bias is likely to be negligible, given the sample size and ratio of ever smokers to never smokers.^23^ Therefore, we consider it unlikely that our single SNP analyses are substantially affected by collider bias.

In conclusion, we found evidence of small effects of genetic liability to smoking initiation and lifetime smoking on increased odds of headache in the last month but we did not find that genetic liability to increased smoking heaviness was associated with headache, suggesting that effects of smoking are related to smoking-related behaviors and may be due to underlying personality factors (eg, impulsivity). Such personality factors may also explain why those with a genetic liability to headaches are less likely to quit. Future work should explore shared risk factors underpinning the risk of both smoking initiation and headache. Future studies should be conducted once larger datasets with more specific headache diagnosis variables are available, and also include adolescent cohorts in whom smoking and headaches are more prevalent. In a clinical setting, health care professionals should be alert to the fact that headache and smoking may share similar risk factors and therefore coexist.

## Supplementary Material

A Contributorship Form detailing each author’s specific involvement with this content, as well as any supplementary data, is available online at https://academic.oup.com/ntr.

ntad173_suppl_Supplementary_Data_S1

ntad173_suppl_Supplementary_Note

ntad173_suppl_Supplementary_Tables_S1-S7

## Data Availability

Researchers can apply for access to UK Biobank, details are available at https://www.ukbiobank.ac.uk/. Genome-wide summary statistics are publicly available, details found in the referenced publications.
