# Supplementary material for: Exploring the Bidirectional Causal Pathways Between Smoking Behaviors and Headache: A Mendelian Randomization Study
Source: Nicotine Tob Res. 2023 Sep 13;26(7):903–12. doi: 10.1093/ntr/ntad173 (PMC11190053; doi:10.1093/ntr/ntad173)
Supplement: ntad173_suppl_Supplementary_Note [file ntad173_suppl_supplementary_note.docx]

Supplementary Note

**Definitions of Covariates from Observational Analyses in UK Biobank**

*Sex.* Participant sex was acquired from NHS records at recruitment, but in some cases updated by participant self-report (data field 31).

*Birthyear.* Participant year of birth was acquired from central registry data and updated by participant self-report where necessary (data field 34).

*Socio-economic position (SEP).* Townsend deprivation index was used as an indicator of SEP (data field 22189). Each participant is assigned a score from the Townsend deprivation index corresponding to the area in which their postcode (immediately prior to joining UK Biobank) is located. Scores of deprivation were derived from four census measures: percentage of households without a car, percentage of overcrowded households, percentage of households not owner-occupied and percentage of persons unemployed.

*Alcohol consumption.* Alcohol intake frequency (data field 1558) was asked to participants as "About how often do you drink alcohol?". Response categories were on a 6 point scale: *Daily or almost daily, three of four times a week, once or twice a week, one to three times a month, special occasions only, never*. Participants who responded “prefer not to answer” were set to missing. If the participant requested more information, they were guided: “If this varies a lot, please provide an average considering your intake over the last year”.

*Sleep duration.* Participants were asked: "About how many hours sleep do you get in every 24 hours? (please include naps)" (data field 1160). If participants answers were less than 1 hour or more than 23 hours then they were set to missing. Participants who responded less than 3 hours or more than 12 hours had their responses confirmed. If the participant requested more information, they were guided: “If the time you spend sleeping varies a lot, give the average time for a 24 hour day in the last 4 weeks.” Participants responding "Do not know" and "Prefer not to answer" were set to missing. Given that participants were asked to confirm extreme answers, we have not excluded any additional outliers.

*Body mass index.* We calculated from participants body mass index (BMI) from their standing height (data field 50) and their weight (data field 21002). BMI was calculated as weight in kg/ height in m^2^. As has been done previously (Di Angelantonio et al., 2016), we excluded participants with a BMI below 15 or above 60.

*Allergic conditions.* Participants were asked: "Has a doctor ever told you that you have had any of the following conditions? (You can select more than one answer)" (data field 6152). Response options were: ‘blood clot in the leg’, ‘blood clot in the lung’, ‘emphysema/chronic bronchitis’, ‘asthma’, ‘hay fever, allergic rhinitis or eczema’, ‘none of the above’ or ‘prefer not to answer’. Participants responding ‘prefer not to answer’ were set to missing. From the remaining responses we created one binary allergy variable, were participants responding yes to asthma, hay fever, allergic rhinitis or eczema were set to 1 and all other responses were set to 0.

*Health care seeking for mental distress.* We combined two variables "Have you ever seen a psychiatrist for nerves, anxiety, tension or depression?" (data field 2100), and "Have you ever seen a general practitioner (GP) for nerves, anxiety, tension or depression?" (data field 2090). Response categories were ‘yes’, ‘no’, ‘don’t know’ and ‘prefer not to answer’. If a participant responded yes to either question then we recorded them as 1. If they responded no to both questions then they were coded as zero. Responses of ‘don’t know’ and ‘prefer not to answer’ responses were set to missing.

**Replication two-sample MR using the FinnGen cohort**

*Sample.* The FinnGen cohort currently comprises almost 300,000 individuals with the ultimate goal of including 500,000 individuals, all of Finnish ancestry (Kurki et al., 2023). The cohort is a public–private partnership, combining imputed genotype data from 9 Finnish biobanks, research institutes, universities and university hospitals, 13 international pharmaceutical industry partners and Finnish registry data. Their nationwide longitudinal health register has been collected since 1969 and captures every resident in Finland. The data is continually being updated to include more sample. We used version R8 of the data, the most recent release. Finland is a population isolate due to a recent bottleneck event in evolutionary history. As a result, deleterious, disease-predisposing alleles can appear at considerably higher frequencies in the Finnish population that older outbred populations, making disease relevant alleles hopefully easier to identify (Kurki et al., 2023).

*Measure.* ‘Other headache Syndromes’ relates to ICD-10 code G44. It includes: cluster headache syndrome, vascular headache, tension-type headache, chronic post-traumatic headache, drug-induced headache, and other specified headache syndromes. It excludes: atypical facial pain, headache not otherwise specified, trigeminal neuralgia and migraines. In the total sample of 278,588 individuals, 13,926 (5.00%) had endorsed this ‘other headache syndromes’ variable. The average age at first event was 48.18 years. The GWAS controlled for sex, age, genotyping batch and ten PCs of population structure (Kurki et al., 2023), and identified 1 genome-wide significant SNP. Additional details about the phenotypes, QC and GWAS procedure are available at <https://finngen.gitbook.io/documentation/>.

*Method.* We estimated causal effects on ‘other headache syndromes’ as the outcome using smoking initiation and lifetime smoking instruments, as described in the main text. Unfortunately, there was only one genome-wide significant SNP identified and therefore we were unable to replicate the bi-directional MR, using this GWAS as the exposure.

*Results.* Two-sample MR results are presented in Supplementary Table S1. Results are consistent for smoking initiation and lifetime smoking when using the IVW and MR Raps methods. Direction of effect was inconsistent for the weighted mode method suggesting possibly more pleiotropy in the FinnGen sample. However, confidence intervals were imprecise, and this method does need the most power.

**References**

Di Angelantonio, E., Bhupathiraju, S. N., Wormser, D., Gao, P., Kaptoge, S., Gonzalez, A. B. de, Cairns, B. J., Huxley, R., Jackson, C. L., Joshy, G., Lewington, S., Manson, J. E., Murphy, N., Patel, A. V., Samet, J. M., Woodward, M., Zheng, W., Zhou, M., Bansal, N., … Hu, F. B. (2016). Body-mass index and all-cause mortality: Individual-participant-data meta-analysis of 239 prospective studies in four continents. *The Lancet*, *388*(10046), 776–786. https://doi.org/10.1016/S0140-6736(16)30175-1

Kurki, M. I., Karjalainen, J., Palta, P., Sipilä, T. P., Kristiansson, K., Donner, K. M., Reeve, M. P., Laivuori, H., Aavikko, M., Kaunisto, M. A., Loukola, A., Lahtela, E., Mattsson, H., Laiho, P., Della Briotta Parolo, P., Lehisto, A. A., Kanai, M., Mars, N., Rämö, J., … Palotie, A. (2023). FinnGen provides genetic insights from a well-phenotyped isolated population. *Nature*, *613*(7944), Article 7944. https://doi.org/10.1038/s41586-022-05473-8
