# Supplementary material for: Exploring the Bidirectional Causal Pathways Between Smoking Behaviors and Headache: A Mendelian Randomization Study
Source: Nicotine Tob Res. 2023 Sep 13;26(7):903–12. doi: 10.1093/ntr/ntad173 (PMC11190053; doi:10.1093/ntr/ntad173)
Supplement: ntad173_suppl_Supplementary_Tables_S1-S7 [file ntad173_suppl_supplementary_tables_s1-s7.docx]

**Supplementary Materials**

**Contents**

| Table S1. Two-sample MR results for the effect of smoking behaviours on risk of headaches in the FinnGen cohort  Table S2. Tests of regression dilution I^2^_GX_ and instrument strength | Page 2  Page 3 |
| --- | --- |
| Table S3. Rucker’s Q tests of heterogeneity in the SNP-exposure effects in MR with summary level data | Page 4 |
| Table S4. MR Egger intercept test for directional pleiotropy | Page 6 |
| Table S5. Testing the direction of association using Steiger filtering | Page 7 |
| Table S6. Baseline characteristics of 336,441 participants in the UK Biobank according to smoking status and number of rs1051730 effect alleles  Supplementary Table S7. MRlap Estimates of Bias from Sample Overlap in Two-Sample MR Analyses | Page 8  Page 9 |
|  |  |
|  |  |

**Table S1.** Two-sample MR results for the effect of smoking behaviours on risk of headaches in the FinnGen cohort

| **Exposure** | **N SNP** | **Method** | **OR (95% CI)** | **P-value** |
| --- | --- | --- | --- | --- |
| **Smoking Initiation** | 235 | Inverse variance weighted | 1.15 (0.992, 1.333) | 0.06 |
|  | 235 | Weighted median | 1.049 (0.848, 1.297) | 0.66 |
|  | 235 | Weighted mode | 0.924 (0.539, 1.584) | 0.77 |
|  | 235 | Robust adjusted profile score (RAPS) | 1.17 (1.002, 1.366) | 0.05 |
| **Lifetime Smoking** | 118 | Inverse variance weighted | 1.43 (1.11, 1.84) | 0.005 |
|  | 118 | Weighted median | 1.33 (0.93, 1.9) | 0.11 |
|  | 118 | Weighted mode | 0.99 (0.40, 2.42) | 0.98 |
|  | 118 | Robust adjusted profile score (RAPS) | 1.43 (1.11, 1.84) | 0.006 |

**Note.** When smoking initiation is the exposure, units have been multiplied by 0.693 so that they can be interpreted as per doubling in the odds of smoking initiation.

**Table S2. Tests of regression dilution I^2^_GX_ and the mean F (mF) statistic which indicates instrument strength**

| **Exposure** | **Unweighted I^2^_GX_** | **Weighted I^2^_GX_** | **mF** |
| --- | --- | --- | --- |
| Smoking initiation -> Headache in the last month | 0.59 | 0.34 | 38.23 |
| Lifetime smoking -> Headache in the last month | 0.64 | 0.40 | 44.05 |
| Smoking initiation -> Headache for more than 3 months | 0.58 | 0.32 | 38.01 |
| Lifetime smoking -> Headache for more than 3 months | 0.64 | 0.41 | 44.05 |
| Headache in the last month -> smoking initiation | 0.79 | 0.75 | 49.25 |
| Headache in the last month -> smoking heaviness | 0.80 | 0.72 | 49.25 |
| Headache in the last month -> smoking cessation | 0.80 | 0.80 | 49.25 |
| Headache for more than 3 months -> smoking initiation | 0.77 | 0.75 | 55.25 |
| Headache for more than 3 months -> smoking heaviness | 0.77 | 0.77 | 55.25 |
| Headache for more than 3 months -> smoking cessation | 0.77 | 0.86 | 55.25 |

**Table S3. Rucker’s Q tests of heterogeneity in the SNP-exposure effects in MR with summary level data**

| **Exposure** | **Outcome** |  | **Q** | **df** | **P-value** |
| --- | --- | --- | --- | --- | --- |
| Smoking initiation | Headache in the last month | Inverse-variance weighted | 566 | 216 | <0.001 |
|  |  | MR Egger | 560 | 215 | <0.001 |
|  |  | Q’ | 6 | 1 | 0.01 |
| Lifetime smoking | Headache in the last month | Inverse-variance weighted | 427 | 119 | <0.001 |
|  |  | MR Egger | 417 | 118 | <0.001 |
|  |  | Q’ | 10 | 1 | 0.002 |
| Smoking initiation | Headache for more than 3 months | Inverse-variance weighted | 406 | 235 | <0.001 |
|  |  | MR Egger | 406 | 234 | <0.001 |
|  |  | Q’ | 0.30 | 1 | 0.58 |
| Lifetime smoking | Headache for more than 3 months | Inverse-variance weighted | 275 | 119 | <0.001 |
|  |  | MR Egger | 268 | 118 | <0.001 |
|  |  | Q’ | 7 | 1 | 0.008 |
| Smoking initiation | Headache (FinnGen) | Inverse-variance weighted | 269 | 234 | 0.06 |
|  |  | MR Egger | 266 | 233 | 0.07 |
|  |  | Q’ | 2 | 1 | 0.13 |
| Lifetime smoking | Headache (FinnGen) | Inverse-variance weighted | 138 | 117 | 0.09 |
|  |  | MR Egger | 136 | 116 | 0.10 |
|  |  | Q’ | 1 | 1 | 0.22 |
| Headache in the last month | Smoking initiation | Inverse-variance weighted | 182 | 26 | <0.001 |
|  |  | MR Egger | 181 | 25 | <0.001 |
|  |  | Q’ | 1 | 1 | 0.27 |
| Headache for more than 3 months | Smoking initiation | Inverse-variance weighted | 63 | 16 | <0.001 |
|  |  | MR Egger | 62 | 15 | <0.001 |
|  |  | Q’ | 1 | 1 | 0.44 |
| Headache in the last month | Smoking heaviness | Inverse-variance weighted | 142 | 26 | <0.001 |
|  |  | MR Egger | 125 | 25 | <0.001 |
|  |  | Q’ | 17 | 1 | <0.001 |
| Headache for more than 3 months | Smoking heaviness | Inverse-variance weighted | 15 | 16 | 0.54 |
|  |  | MR Egger | 11 | 15 | 0.77 |
|  |  | Q’ | 4 | 1 | 0.04 |
| Headache in the last month | Smoking cessation | Inverse-variance weighted | 54 | 26 | 0.001 |
|  |  | MR Egger | 54 | 25 | <0.001 |
|  |  | Q’ | 0.02 | 1 | 0.89 |
| Headache for more than 3 months | Smoking cessation | Inverse-variance weighted | 14 | 16 | 0.63 |
|  |  | MR Egger | 13 | 15 | 0.59 |
|  |  | Q’ | 0.35 | 1 | 0.55 |

**Note.** The Q statistic measures for the IVW method estimates the amount of heterogeneity when we assume that all of the SNPs are valid instruments. Therefore, heterogeneity here could indicate horizontal pleiotropy but also vertical pleiotropy which does not bias the MR estimates. The Q statistic measures for the MR Egger method assesses heterogeneity after fitting the MR-Egger regression model and adjusting for the mean pleiotropic effect. Therefore, it is possible to test whether any residual heterogeneity due to pleiotropy remains. Therefore, if the Q difference between the two Q statistics is significant, then this could suggest that the IVW estimate is biased by horizontal pleiotropy which has been accounted for with MR Egger.

**Table S4. MR Egger intercept test for directional pleiotropy**

| **Exposure** | **Outcome** | **Intercept (95% CI)** | **P-value** |
| --- | --- | --- | --- |
| Smoking initiation | Headache in the last month | 0.001 (-0.0003, 0.003) | 0.13 |
| Lifetime smoking | Headache in the last month | 0.002 (-0.0003, 0.004) | 0.10 |
| Smoking initiation | Headache for more than 3 months | -0.0001 (-0.0008, 0.0005) | 0.68 |
| Lifetime smoking | Headache for more than 3 months | 0.0008 (-0.0001, 0.002) | 0.08 |
| Smoking initiation | Headache (FinnGen) | 0.008 (-0.003, 0.019) | 0.16 |
| Lifetime smoking | Headache (FinnGen) | 0.009 (-0.007, 0.024) | 0.27 |
| Headache in the last month | Smoking initiation | 0.001 (-0.006, 0.009) | 0.69 |
| Headache for more than 3 months | Smoking initiation | -0.001 (-0.009, 0.006) | 0.71 |
| Headache in the last month | Smoking heaviness | 0.008 (0.000, 0.016) | 0.08 |
| Headache for more than 3 months | Smoking heaviness | 0.006 (0.000, 0.011) | 0.06 |
| Headache in the last month | Smoking cessation | 0.0002 (-0.004, 0.004) | 0.92 |
| Headache for more than 3 months | Smoking cessation | -0.001 (-0.006, 0.003) | 0.56 |

**Table S5. Testing the direction of association using Steiger filtering**

| **Exposure** | **Outcome** | **N SNP** |
| --- | --- | --- |
| Smoking initiation | Headache in the last month | 217/217 (100%) |
| Lifetime Smoking | Headache in the last month | 120/120 (100%) |
| Smoking initiation | Headache for more than 3 months | 236/236 (100%) |
| Lifetime Smoking | Headache for more than 3 months | 120/120 (100%) |
| Headache in the last month | Smoking initiation | 17/28 (61%) |
| Headache for more than 3 months | Smoking initiation | 10/27 (59%) |
| Headache in the last month | Smoking heaviness | 18/28 (64%) |
| Headache for more than 3 months | Smoking heaviness | 11/17 (65%) |
| Headache in the last month | Smoking cessation | 22/28 (79%) |
| Headache for more than 3 months | Smoking cessation | 11/17 (65%) |

**Table S6. Baseline characteristics of 336,441 participants in the UK Biobank according to smoking status and number of rs1051730 effect alleles**

|  |  | Never smokers | | | |  | |  | |  | | Ever smokers | | | |  | |  | |
| --- | --- | --- | --- | --- | --- | --- | --- | --- | --- | --- | --- | --- | --- | --- | --- | --- | --- | --- | --- |
|  |  | **0** | | **1** | | **2** | | **p-value** | |  | | **0** | | **1** | | **2** | | **p-value** | |
| Study sample (n) |  | 82,017 | | 81,436 | | 20,300 | |  | |  | | 68,444 | | 66,819 | | 16,278 | |  | |
| Cigarettes per day (mean) | |  |  | |  | |  | |  | | 14.87 | | 15.90 | | 16.72 | | <0.001 | |  |
| Missing (n) |  |  | |  | |  | |  | |  | | 3800 | | 3362 | | 736 | |  | |
| Smoking status (%)^a^ |  |  | |  | |  | |  | |  | |  | |  | |  | |  | |
| Current |  |  | |  | |  | |  | |  | | 22 | | 22 | | 22 | |  | |
| Former |  |  | |  | |  | |  | |  | | 78 | | 78 | | 78 | | 0.064 | |
| Men (%) |  | 42 | | 41 | | 41 | | 0.131 | |  | | 52 | | 52 | | 52 | | 0.552 | |
| Age (mean) |  | 56.1 | | 56.2 | | 56.2 | | 0.094 | |  | | 57.8 | | 57.7 | | 57.6 | | 0.002 | |
| Education (%) | |  |  | |  | |  | |  | |  | |  | |  | |  | |  |
| Primary |  | 14 | | 14 | | 14 | |  | |  | | 21 | | 21 | | 20 | |  | |
| Secondary |  | 51 | | 50 | | 50 | |  | |  | | 51 | | 52 | | 52 | |  | |
| Tertiary |  | 36 | | 36 | | 36 | | 0.805 | |  | | 27 | | 27 | | 28 | | 0.018 | |
| Missing (n) |  | 672 | | 690 | | 198 | |  | |  | | 626 | | 672 | | 131 | |  | |
| Alcohol intake (%) |  |  | |  | |  | |  | |  | |  | |  | |  | |  | |
| Never |  | 7 | | 7 | | 7 | |  | |  | | 5 | | 6 | | 6 | |  | |
| Special occasions |  | 11 | | 12 | | 11 | |  | |  | | 9 | | 9 | | 9 | |  | |
| Monthly |  | 12 | | 13 | | 12 | |  | |  | | 9 | | 10 | | 10 | |  | |
| Weekly |  | 52 | | 53 | | 53 | |  | |  | | 49 | | 47 | | 47 | |  | |
| Daily/almost daily |  | 17 | | 16 | | 16 | | 0.173 | |  | | 28 | | 28 | | 28 | | 0.132 | |
| Missing (n) |  | 32 | | 42 | | 15 | |  | |  | | 46 | | 53 | | 12 | |  | |
| Troubled by headaches past month (%) |  | 20.6 | | 20.9 | | 20.0 | | 0.008 | |  | | 18.9 | | 19.1 | | 18.7 | | 0.416 | |
| Troubled by headaches more than 3 months (%) |  | 9.37 | | 9.60 | | 9.26 | | 0.174 | |  | | 8.30 | | 8.42 | | 8.28 | | 0.707 | |

**Supplementary Table S7.** MRlap Estimates of Bias from Sample Overlap in Two-Sample MR Analyses

| **Exposure** | **Outcome** | **Method** | **N SNPs** | **OR (95% CI)** | **P-value** |
| --- | --- | --- | --- | --- | --- |
| Smoking Initiation | Headache in the last month | IVW | 225 | 1.10 (1.04, 1.16) | <0.001 |
|  |  | Corrected |  | 1.11 (1.04, 1.18) | <0.001 |
|  |  | Difference |  | -1.96 | 0.05 |
| Lifetime Smoking | Headache in the last month | IVW | 126 | 1.17 (1.09, 1.25) | <0.001 |
|  |  | Corrected |  | 1.20 (1.10, 1.31) | <0.001 |
|  |  | Difference |  | -2.59 | 0.01 |
| Smoking Initiation | Headache for more than 3 months | IVW | 239 | 1.004 (0.97, 1.04) | 0.80 |
|  |  | Corrected |  | 1.007 (0.97, 1.05) | 0.74 |
|  |  | Difference |  | -0.56 | 0.58 |
| Lifetime Smoking | Headache for more than 3 months | IVW | 126 | 1.04 (1.00, 1.09) | 0.07 |
|  |  | Corrected |  | 1.06 (1.03, 1.09) | 0.05 |
|  |  | Difference |  | -2.21 | 0.03 |

Note. IVW = inverse variance weighted. Corrected = IVW MR result corrected for bias from sample overlap. Therefore, if the difference between these two estimates is significant, then this suggests that the MR results were biased by sample overlap.
